# Diagnostic challenges and therapeutic approaches in pediatric osteosarcoma: A case report

**DOI:** 10.1016/j.ijscr.2025.111815

**Published:** 2025-08-14

**Authors:** Jlalia Zied, Mansi Zied, Arfa Wajdi, Ben salah Jihed, Guedhami Hedhili, Souid Abderrahmen

**Affiliations:** a Orthopedic Pediatric Department, Kassab Institute of Orthopedic Surgery, Tunisia; b Department of Orthopedic Surgery, IBN EL JAZZAR University Hospital, Kairouan, Tunisia; c Department of Orthopedic Surgery, Hospital of Gafsa, Gafsa, Tunisia

**Keywords:** Osteosarcoma (OS), Diagnosis, Radiology, Chemotherapy, Induced membrane technique, Bone reconstruction

## Abstract

**Introduction and importance:**

Osteosarcoma (OS) is the most common primary malignant bone tumor. Radiological features may resemble benign processes, making diagnosis challenging. Delayed recognition can affect treatment outcomes, as timely intervention is crucial for managing the tumor effectively.

**Case presentation:**

This report discusses the case of a 9-year-old girl who presented with left knee pain, without signs of fever or inflammation. Radiological imaging revealed a large, multilocular osteolytic lesion in the distal femur with sclerotic margins and no periosteal reaction, initially suggesting subacute osteomyelitis. A biopsy confirmed the diagnosis of OS. Staging studies revealed no distant metastases. The patient underwent neoadjuvant chemotherapy followed by a two-stage surgical procedure using the induced membrane technique for bone reconstruction. Histological examination of the resected tumor showed a high-grade osteoblastic OS with a good response to chemotherapy. After adjuvant chemotherapy, the patient achieved complete remission. One year postoperatively, a non-vascularized autograft was performed using fibula and iliac grafts.

**Discussion:**

The induced membrane technique proved successful, with good functional results and no complications. This case highlights the diagnostic challenges of osteosarcoma, particularly when the radiological features resemble benign conditions. Early diagnosis and a multidisciplinary approach, including surgery and chemotherapy, are essential for improving patient outcomes. Follow-up is critical to detect late complications in OS survivors.

**Conclusion:**

Pediatric osteosarcoma presents significant diagnostic and therapeutic challenges. Early detection and a combination of surgery and chemotherapy are key to improving outcomes.

## Highlights

• The interest of this case is to show the technique of epiphyseal preservation during bone resection of an osteosarcoma of the distal femur in a child.
• This case highlights the diagnostic challenges of osteosarcoma, particularly when the radiological features resemble benign conditions
• Early diagnosis and a multidisciplinary approach, including surgery and chemotherapy, are essential for improving patient outcomes. Follow-up is critical to detect late complications in osteosarcoma survivors.

## 1 Introduction

Osteosarcoma (OS) is the most commonly diagnosed primary malignant bone tumor most commonly affecting children and young adults, with a global incidence of 3.4 cases per million people annually [1,2]. It represents twice as much as chondrosarcoma [3]. Their preferred locations are the metaphysis of the following long bones: the distal femur (40 %), proximal tibia (16 %) and proximal humerus (15 %) [3].

It is often characterized by its ability to produce osteoid (bone-like tissue) and can lead to a variety of radiological appearances, which may mimic other benign or malignant conditions, resulting in a diagnostic challenge [4]. It typically presents radiologically as an aggressive, destructive lesion characterized by a mixed osteolytic and sclerotic pattern, often accompanied by periosteal reactions and soft-tissue masses [5,6]. While osteosarcoma is most often associated with these aggressive features, purely osteolytic lesions are relatively uncommon, accounting for only about 10 % of cases. These osteolytic variants are typically found in subtypes such as telangiectatic, fibroblastic, malignant fibrous histiocytoma-like, giant cell-rich, or low-grade OS, and are most frequently observed in elderly patients [7,8].

Through this clinical case, we will emphasize the diagnostic challenges of osteosarcomas and possible errors, as well as therapeutic modalities.

## 2 Methods

The work has been reported in line with the SCARE criteria [18].

## 3 Case presentation

We present the case of a 9-year-old girl, without medical history, consulted with a major complaint of left knee pain lasting for 1 month. There was neither fever nor biological inflammatory.

**Fig. 1:**
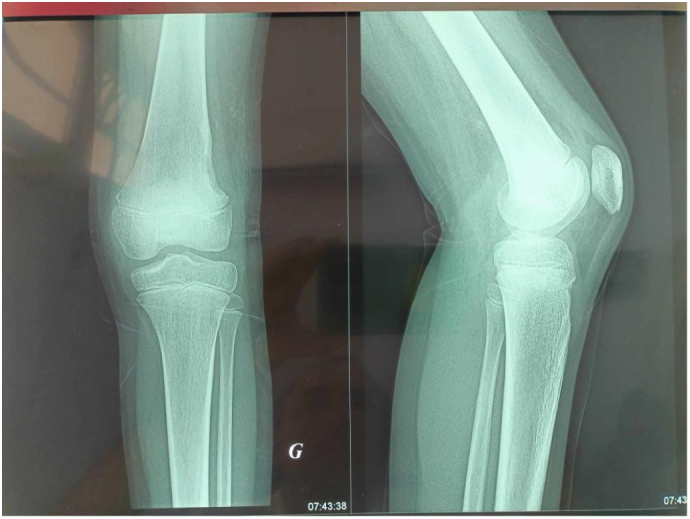
Anteroposterior and lateral radiographs of the knee reveal a well-defined osteolytic lesion measuring 5 cm, located in the external cortex of the distal femoral metaphysis. The lesion exhibits a sclerotic margin, a narrow zone of transition, and no evidence of periosteal reaction.

**Fig. 2:**
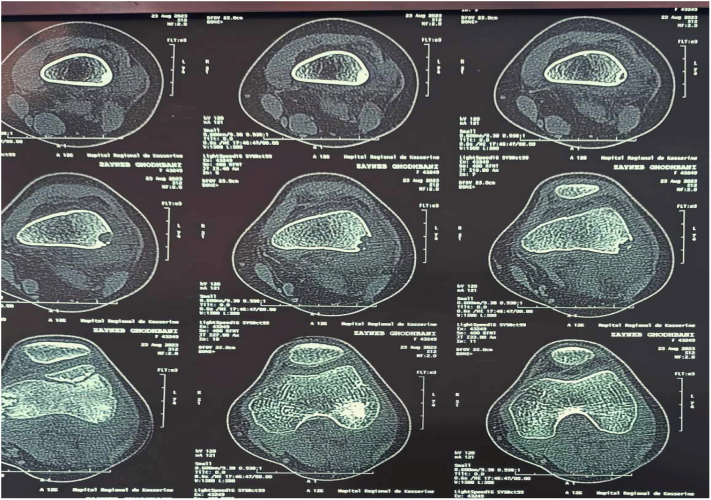
CT scan: osteolytic lesion with clear boundaries with postero-external cortical break-in.

**Fig. 3:**
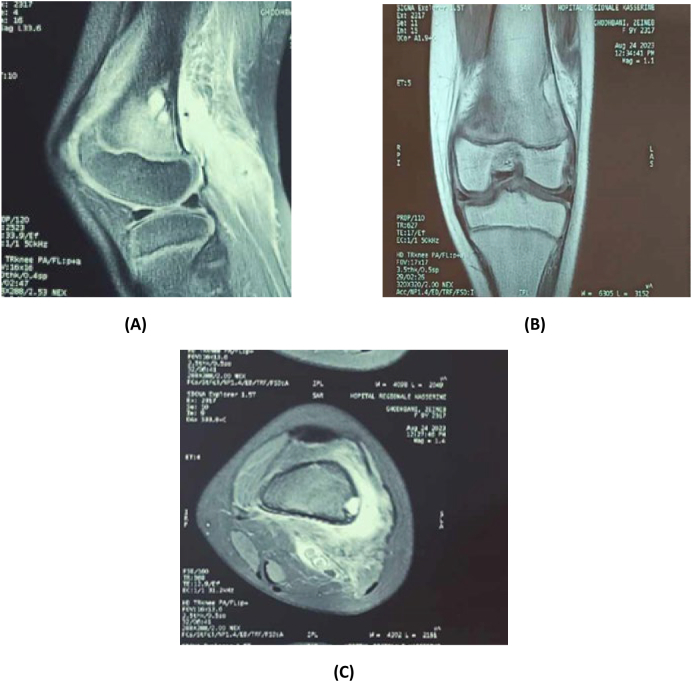
Knee MRI: A and C: T1 MRI sequency B: T2 MRI sequency MRI revealed a multilocular cystic lesion measuring 5 cm, arising from the lateral cortex of the distal femoral metaphysis, with no evidence of soft tissue extension.

Radiographic evaluation revealed a large, multilocular osteolytic lesion involving the external cortex of the distal femoral metaphysis. The lesion appeared well-circumscribed, with sclerotic margins and endosteal scalloping, and showed no evidence of periosteal reaction, consistent with a Lodwick Type I pattern suggestive of a benign and non-aggressive process (Fig. 1). Computed tomography (CT) further delineated the lesion as a well-defined osteolytic area with a postero-external cortical breach (Fig. 2). Due to diagnostic uncertainty, magnetic resonance imaging (MRI) of the knee was performed, revealed a multilocular cystic lesion measuring 5 cm, arising from the lateral cortex of the distal femoral metaphysis, with no evidence of soft tissue extension or periosteal reaction (Fig. 3).

Radiological signs were in favor of sub-acute osteomyelitis.

**Fig. 4:**
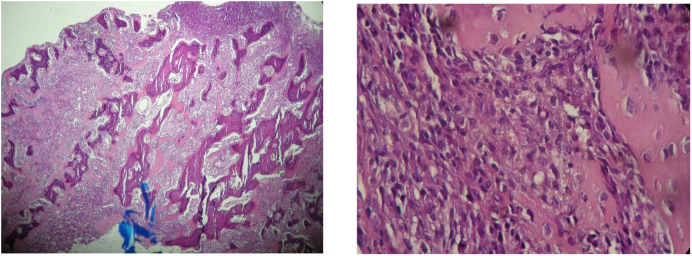
Histological diagnosis: conventional osteosarcoma with soft tissue and synovium damage.

The girl was then operated through a lateral approach, we found tumor tissue which we biopsied. The histological diagnosis was osteoblastic OS with soft tissue and synovium damage (Fig. 4).

An extension assessment was carried out including a chest CT scan, an abdominal ultrasound and a bone scan showing no loco-regional or distant metastases.

The patient underwent surgery and tumor resection after neoadjuvant chemotherapy. Indeed, Chemotherapy was administered, and a follow-up MRI showed a significant reduction in the tumor size. Following this, the patient underwent a two-stage surgical procedure using the induced membrane technique.

**Fig. 5:**
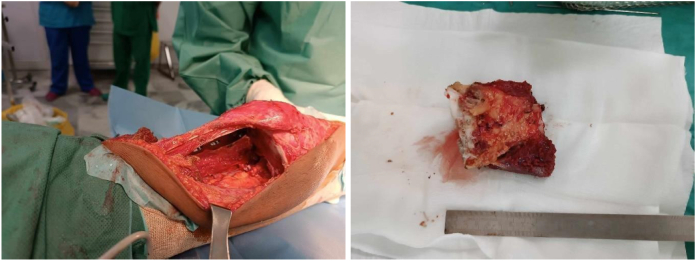
En-bloc intercalary resection, the distal femoral osteotomy was trans-epiphyseal and 2 mm from the epiphyseal invasion.

Initially, en-bloc intercalary resection was performed through lateral approach with epiphyseal preservation. The distal femoral osteotomy was trans-epiphyseal and 2 mm from the epiphyseal invasion (Fig. 5).

**Fig. 6:**
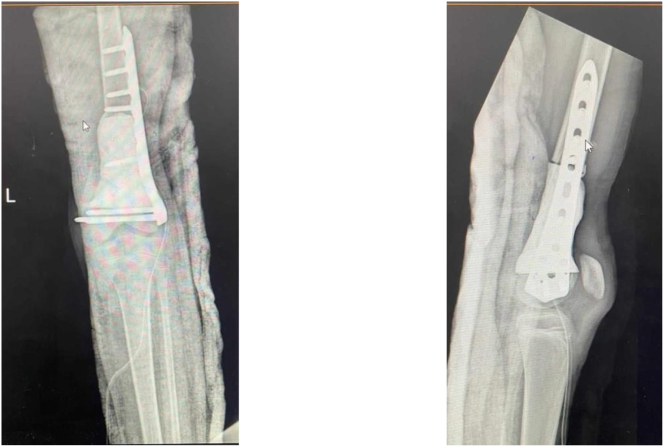
Post-operative X-ray: Bone reconstruction used spacer and stabilization using an anatomical plate.

Polymethyl methacrylate spacer was used for bone reconstruction and initial stabilization was obtained using an anatomical plate (Fig. 6). The length of the bone defect after resection was 7.5 cm.

Histological study of the resected specimen showed a cystic tumor with safe surgical margins and good response to chemotherapy (3 % viable cells). Furthermore, it confirmed the diagnosis of high-grade osteoblastic OS. Final diagnosis was conventionnal OS.

Then, adjuvant chemotherapy was carried out with a good clinical evolution and complete remission.

**Fig. 7:**
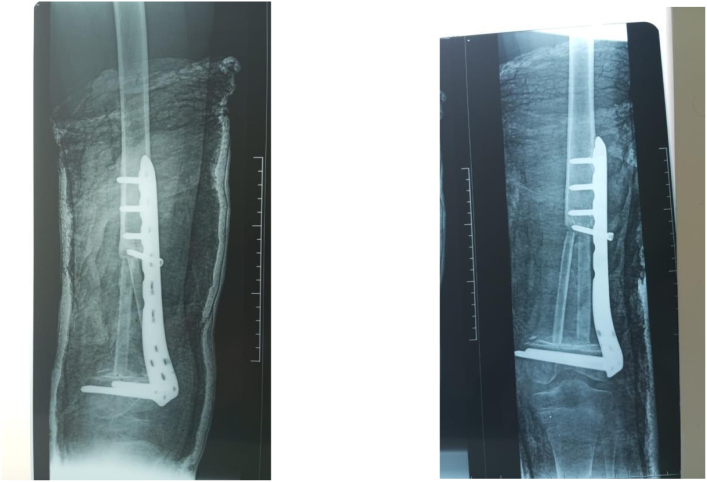
Non-vascularized autograft (two fibulas and cancellous iliac graft).

**Fig. 8:**
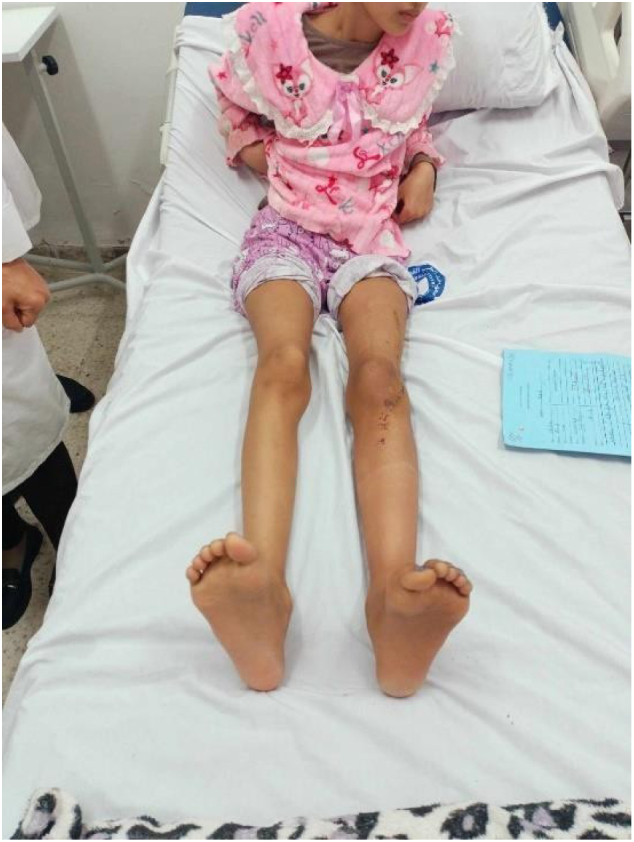
Good clinical outcomes.

One year later after postoperative chemotherapy, a non-vascularized autograft (two fibulas and cancellous iliac graft) was done (Fig. 7, Fig. 8).

**Fig. 9:**
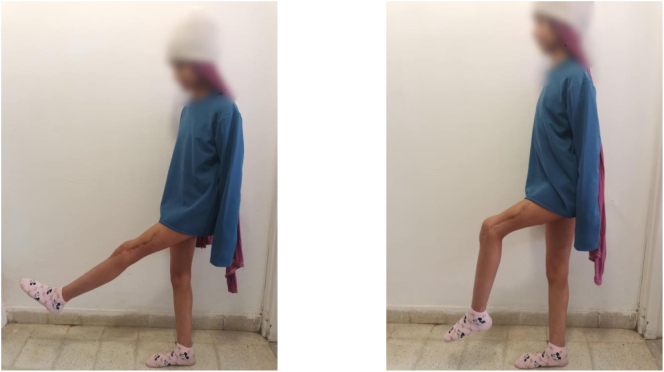
After one year of follow-up; full extension of the knee with 90° of active flexion.

**Fig. 10:**
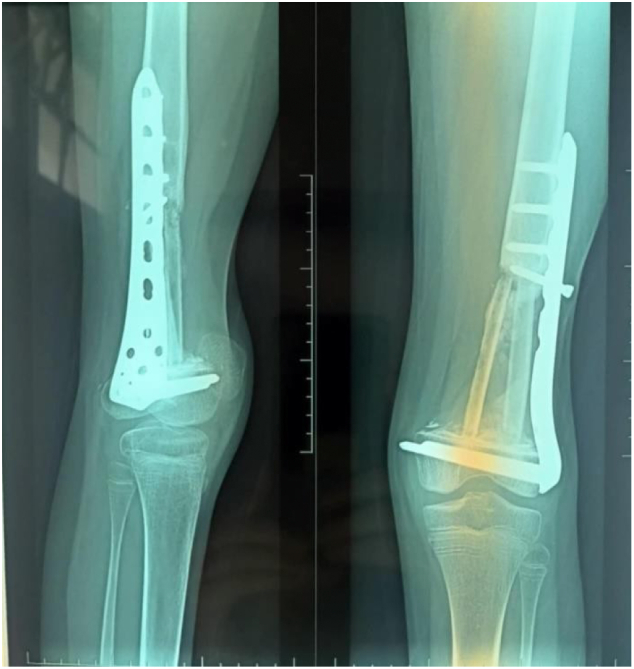
Radiological result after a one-year follow-up.

At one-year follow-up, there was no sign of tumor recurrence, with successful graft consolidation and favorable functional results (Fig. 9, Fig. 10).

## 4 Discussion

Osteosarcomas are the most common non-hematologic primary malignant tumors of bone in adolescents and young adults with an average age between 10 and 30 years [9], comprising about 15 % of all primary bone tumors [1]. The incidence of osteosarcoma has been relatively stable over the last 40–50 years while mortality rates have decreased, primarily due to the introduction of multiagent chemotherapy [10]. Osteosarcomas are most commonly located in the metaphysis of long bones, especially around the knee in the distal femur or proximal tibia [[1], [2], [3]].

It is derived from bone-forming mesenchymal cells. Genomic alterations in TP53, and specifically TP53 inactivation, as well as RB inactivation are present in most osteosarcoma cases. OS are most commonly located in the metaphysis of long bones, especially around the knee in the distal femur or proximal tibia. Similar to Ewing sarcoma, osteosarcoma is most commonly diagnosed in adolescent males, and up to 25 % of patients will demonstrate evidence of metastatic disease at diagnosis with lung metastases being the most common site [11].

Osteosarcoma (OS), the most common primary malignant bone tumor in children and adolescents, typically presents with localized pain and swelling near the metaphyseal regions of long bones. The pain is characteristically dull, progressive, and often worse at night. Swelling may be visible or palpable, and in certain cases, the first clinical manifestation may be a pathological fracture due to tumor-induced bone fragility.

Radiographically, OS often demonstrates a mixed lytic-sclerotic pattern, reflective of simultaneous bone destruction and neoplastic osteoid formation. This heterogeneous appearance is typical of conventional OS, while purely osteolytic variants—lacking sclerotic components—are rare, accounting for approximately 10 % of cases [6]. These atypical radiological features can contribute to misinterpretation and diagnostic delays.

The diagnosis of osteosarcoma is frequently challenging due to its variable clinical and radiographic presentation. In its early stages, OS may lack classic aggressive imaging features such as periosteal reaction (e.g., Codman's triangle or sunburst pattern), cortical breach, or soft tissue extension. These subtle or absent radiologic signs can result in confusion with benign lesions, including simple bone cysts, subacute osteomyelitis, eosinophilic granuloma, or aneurysmal bone cysts.

Advanced imaging modalities such as magnetic resonance imaging (MRI) and computed tomography (CT) are essential for evaluating tumor extent, medullary involvement, and soft tissue extension. However, imaging alone is rarely sufficient for a definitive diagnosis. Histopathological confirmation remains the gold standard and is essential for distinguishing osteosarcoma from other malignant bone tumors, such as Ewing sarcoma or chondrosarcoma. Diagnostic accuracy may be compromised by suboptimal biopsy techniques, including non-representative sampling or incisions that compromise future limb-salvage options.

The clinical implications of diagnostic delays or misdiagnosis are significant. Inaccurate diagnosis can lead to inappropriate or delayed treatment, increased risk of metastasis, diminished opportunities for limb-salvage procedures, and worse oncologic outcomes. Conversely, overdiagnosis may result in unnecessary exposure to chemotherapy or radical surgery. Consequently, early referral to specialized sarcoma centers and a multidisciplinary approach—incorporating orthopedic oncologists, radiologists, and pathologists—is critical to optimize diagnosis and treatment.

A study by Sundaram et al. [6] exemplifies these challenges. They described four cases of osteosarcoma with atypical radiological features: each lesion was confined to a single bone compartment, exhibited a purely osteolytic pattern, and lacked both periosteal reaction and soft tissue mass. The initial radiologic interpretation in all cases was benign—either a simple bone cyst or aneurysmal bone cyst. In our case, the lesion was initially mistaken for subacute osteomyelitis. Definitive diagnosis was only achieved through histopathological analysis, which revealed conventional OS.

Notably, one of Sundaram et al.'s cases was initially diagnosed histologically as an aneurysmal bone cyst. Only after the patient developed pulmonary metastases was a re-biopsy performed, revealing coexistent areas of conventional osteosarcoma within the lesion. Furthermore, two of their cases involved uncommon tumor localizations: one in the femoral head and neck, and another in the tarsal navicular bone. Osteosarcoma in the femoral head and neck accounts for less than 1 % of cases [10], while OS of the foot bones comprises approximately 0.8 % [4].

These findings highlight the critical importance of considering osteosarcoma in the differential diagnosis of atypical bone lesions, particularly when imaging findings are ambiguous or the lesion is located in an uncommon site. A high index of suspicion, appropriate imaging, and accurate biopsy planning are essential to avoid diagnostic errors. Ultimately, multidisciplinary collaboration remains the cornerstone of early detection and successful management of osteosarcoma.

A multidisciplinary team, including surgeons, oncologists, radiologists, and pathologists, works collaboratively to ensure the surgery is thorough and effective, with the ultimate aim of providing the best chance for long-term survival while maintaining the patient's quality of life.

For osteosarcoma patients, the relative 5-year survival rate was 60 % for those younger than 30 years, 50 % for those aged 30 to 49 years, and 30 % for those aged 50 years or older [1].

Curative therapy of high-grade osteosarcoma consists of chemotherapy and surgery. Comparing to surgery alone multimodal chemotherapy increased disease-free survival probability from 10 %–20 % to >60 %. In European protocols and guidelines, chemotherapy is used before and after surgery [11].

Osteosarcoma surgery must be carefully planned and performed with an oncological approach to ensure the complete removal of the tumor while preserving healthy surrounding tissue. The primary goal is to achieve negative resection margins, meaning to reduce the risk of recurrence.

Radiation therapy is reserved in the adjuvant setting for cases with microscopic positive margins or incomplete resection or in the definitive setting for tumors not amenable to complete resection [12].

After tumor removal, bone reconstruction is necessary [13]. This can be done using various methods, including vascularized fibular grafts, segmental allografts, extra-corporeally irradiated autografts, bone transport, chondrodiastasis, or reconstruction prostheses. However, each of these techniques carries potential complications [13]. The induced-membrane technique, developed by Masquelet et al. [14], enables two-stage reconstruction of long bones and has shown promising results in patients, including children, with bone defects caused by trauma, infection, or bone cancer resection.

This technique has several advantages:

• It allows for the reconstruction of segmental bone defects larger than 15 cm, enhancing the likelihood of complete tumor resection.
• The procedure is quick, safe, and does not require specialized equipment, minimizing the risk of long-term complications.
• Surgeons of varying experience can perform the procedure easily, regardless of the resection time, due to the short duration of the reconstruction.

However, it is recommended to delay the implantation of a bone graft until after the completion of adjuvant chemotherapy. This is because chemotherapy agents can negatively affect osteoblast function and cause neutropenia, which increases the risk of infection [15,16].

The primary complication associated with the induced membrane technique was fracture of the reconstructed bone with large defects [16,17]. Another reported complication is massive graft resorption after femur reconstruction [17].

In our case, we have used this technique successfully without any complication and with good functional results. This follow-up is short to detect late complications, which may arise in OS survivors. These patients should be controlled with longer follow-up.

While managing bone reconstruction and its associated challenges is essential, ensuring an accurate diagnosis is equally critical. The article highlights that in patients experiencing persistent bone pain with purely lytic lesions and no aggressive imaging features, clinicians must maintain a high suspicion for osteosarcoma—even when initial imaging or biopsy suggests a benign condition. Early referral to a specialized tumor center and meticulous biopsy planning are vital to avoid misdiagnosis and to provide patients with the best chance for curative treatment.

## 5 Conclusion

In conclusion, osteosarcoma presents a significant diagnostic challenge due to its non-specific symptoms, variable radiographic appearance, and histopathological diversity. Early detection is often hindered by the resemblance to other musculoskeletal conditions, and its diagnosis typically requires a comprehensive approach involving imaging techniques, biopsy, and sometimes genetic testing. Given the potential for misdiagnosis and the aggressive nature of the disease, a high index of suspicion is crucial for timely and accurate diagnosis, ultimately leading to better treatment outcomes for patients.

## Author contribution

Dr. Jlalia Zied: study concept, data collection, interpretation, Supervision, Validation

Dr. Mansi Zied: analysis, data collection, writing the paper

Dr. Arfa Wajdi: analysis and data collection

Dr. Ben salah Jihed: analysis and data collection

Dr. Guedhami Hedhili: study concept and data collection

Dr. Souid Abderrahmen: interpretation, Validation

## Consent

Written informed consent was obtained from the patient's parents/legal (the father) guardian for publication and any accompanying images. A copy of the written consent is available for review by the Editor-in-Chief of this journal on request.

## Ethical approval

Ethical approval for this study (ethical approval number N°25) was provided by the Ethical Committee of IBN EL JAZZAR Hospital, Kairouan, Tunisia on 10 Mai 2024.

## Guarantor

Dr: MANSI ZIED.

## Research registration number

1023589

## Funding

There is no source of funding for our research.

## Conflict of interest statement

The authors declare that they have no known competing financial interests or personal relationships that could have appeared to influence the work reported in this paper.
